# Use of Smartphone Health Apps Among Patients Aged 18 to 69 Years in Primary Care: Population-Based Cross-sectional Survey

**DOI:** 10.2196/34882

**Published:** 2022-06-16

**Authors:** Sabrina Paradis, Jeremy Roussel, Jean-Luc Bosson, Jean-Baptiste Kern

**Affiliations:** 1 Department of General Practice University Grenoble Alpes Grenoble France; 2 Translational Innovation in Medicine and Complexity National Institute of Health and Medical Research La Tronche France; 3 Department of Public Health University Grenoble Alpes Grenoble France

**Keywords:** smartphone, health applications, mHealth, apps, mobile health, digital health, well-being, epidemiology, primary care, population survey, fitness, physical activity, health behavior, patient

## Abstract

**Background:**

The World Health Organization has defined mobile health (mHealth) as the “use of mobile and wireless technologies to support the achievement of health objectives.” Smartphones currently represent one of the main media forms for mHealth democratization. Health apps can be an interesting tool for changing health behaviors. However, their use in France is still poorly documented.

**Objective:**

The main aim of this study was to evaluate the frequency of use of health apps among patients consulting in the primary care setting in France. The secondary aims were to evaluate the use of health apps according to the sociodemographic and medical characteristics of patients and to determine their use.

**Methods:**

A population-based cross-sectional survey was carried out between November 2017 and January 2018 in the Grenoble area of France among patients aged between 18 and 69 years who were consulting at 13 primary care physician offices. Patients were provided with anonymous paper self-questionnaires. The main criterion for participation was the use of a smartphone health app, defined for the purpose of this study as any app supporting patients in efforts to be healthy.

**Results:**

The participation rate was 49.27% (739/1500; 95% CI 46.7%-51.8%). The smartphone use was estimated at 82.6% (597/723; 95% CI 79.6%-85.2%). Of 597 smartphone owners, 47.7% (283/595; CI 43.6%-51.6%) used at least one smartphone health app. Health apps identified in this study were mainly related to wellness, prevention, and fitness (66.1%), as well as medication, treatments, and follow-up care (50.0%). The main factors associated with health app use were: use of social networks (odds ratio [OR] 3.4, 95% CI 2.1-5.3), age under 30 years (OR 2.7, CI 1.4-4.9), city size between 5001 and 10,000 inhabitants (OR 1.8, CI 1.1-2.8), and city size more than 10,000 inhabitants (OR 2.1, CI 1.4-3.2).

**Conclusions:**

In this survey, nearly one out of two patients reported the use of smartphone health apps, which are currently focused on wellness, prevention, and fitness, and are largely used by the younger population.

**Trial Registration:**

ClinicalTrials.gov NCT03351491; https://clinicaltrials.gov/ct2/show/NCT03351491

## Introduction

Mobile health (mHealth) is generally defined as medical and public health practice supported by mobile devices such as mobile phones, patient monitoring devices, personal digital assistants, and other wireless devices. The World Health Organization has defined mHealth as the “use of mobile and wireless technologies to support the achievement of health objectives” [1].

A mobile phone is a wireless portable device that allows users to make and receive calls. Modern mobile phones are more commonly called “smartphones” because of the many digital mobile services they offer. Owing to their advanced computing and their connectivity using cellular network architecture, smartphones currently represent one of the main media forms for mHealth democratization and spread. Between 2011 and 2020 in France, smartphone ownership among people older than 12 years increased from 17% to 84%, with 94% of the population older than 12 years owning a mobile phone in 2020 [2]. Similar statistics were reported the same year in the United States, with a rate of smartphone ownership estimated at 85% [3]. This equipment evolution has been accompanied by an increase in the number and use of mobile apps. These software programs running on devices such as smartphones are preinstalled or downloadable on apps markets (eg, Google Play Store, Apple App Store). Some of these focus on health, fitness, or medical care.

In the last few decades, smartphones have radically modified our daily lives. In the field of health, patients have more access to health knowledge, with greater opportunities to improve the involvement of patients through patient-professional partnerships. Several studies have demonstrated the effectiveness of mHealth interventions to improve health behavior [4,5] in contexts such as diet adhesion, smoking cessation, increasing physical activity, and chronic disease management (eg, diabetes or hypertension). This evolution has impacted the relationship between general practitioners (GPs) and their patients [6]. Recent research suggests that patients perceive mHealth apps as useful complementary tools for self-monitoring and self-management of their health, albeit with some limits [7]. A qualitative study involving French GPs highlighted an ambivalent discourse around the prescription of mHealth apps or patients’ use of apps [8]. In France, health apps must be considered as “medical devices” and must be evaluated by the National Committee for the Evaluation of Medical Devices and Health Technologies (CNEDIMTS) to obtain reimbursement [9]. Currently, to our knowledge, only one app is reimbursed by the health insurance and can be prescribed (Moovcare).

In 2015, a survey conducted in the United States showed that 58.2% of mobile phone users had a health app compared to only 19% in 2012 [10]. The proportion of smartphone owners using health apps was estimated at 20.5% in 2015 in Germany [11] and at 24.1% in 2016 in Hong Kong [12]. However, there is a paucity of data in the scientific literature concerning the use of health apps among smartphone users in France.

The main objective of this study was to evaluate the frequency of use of at least one mobile health app on a smartphone since its acquisition by patients recruited from the primary care setting in the Grenoble region of France. The secondary objectives were to collect the types of health apps used, and to analyze factors associated with the use of these apps according to sociodemographic, geographical, and medical characteristics of the studied population.

## Methods

### Design

This study was a population-based cross-sectional survey carried out among patients aged 18-69 years consulting GPs in their office or in a primary care center in the Grenoble area of France. Data were collected between November 2017 and January 2018 by anonymous self-administered paper questionnaires.

### Sample

Inclusion criteria of the considered sample were: all adult outpatients aged between 18 and 69 years, consulting a GP and not being deprived of liberty by judicial or administrative decision or subject to a legal protection measure. All patients were eligible regardless of the reason for the consultation. The sample size was calculated using the following assumptions: precision=5%, α risk=5%, estimated percentage of smartphone users=65%, estimated health apps use in the population of smartphone users=50%. The calculated number was then increased by 60% to take nonresponses into account. The minimum required sample size was estimated at 1476 questionnaires. This number was then rounded up to 1500.

### Questionnaire

A specific questionnaire was developed for the purpose of the study and distributed to participants. The questionnaire consists of 23 questions and was estimated to be completed in approximately 5 minutes. It was submitted to expert opinion (one GP, one epidemiologist, and one biostatistician) and then tested on 15 patients. Their advice and suggestions were taken into account, and the questionnaire was modified to be as understandable and relevant as possible. As health apps may be more frequently used by internet users, we opted for a paper questionnaire to avoid potential selection bias. After receiving clear oral information on the terms and objectives of the study, written information was presented on the first page. A patient’s refusal to participate in the study could be indicated on the first page. A total of 1500 self-administered anonymous paper questionnaires were distributed to 35 voluntary GPs in 12 offices and in 1 primary care center. These were selected to obtain a representative sample of patients in terms of sociodemographic factors (see Multimedia Appendix 1). Convenience sampling was applied in the centers. Questionnaires were administered by the GPs or their secretaries after having checked that the inclusion criteria were respected. Once completed, they were stocked in a box in each investigation center. All boxes were collected during the last week of January 2018.

The first question collected the details of mobile phone and smartphone equipment owned. The primary outcome measure was the use of at least one mobile health app on a smartphone. In this study, a health app refers to an app supporting patients in efforts to be healthy without distinguishing between wellness and prevention apps. Online health and well-being websites available with a smartphone were also considered to be in scope. The download and the use of smartphone apps were also measured without distinguishing between common apps and health apps. The secondary outcome measures were the digital characteristics (determined by the digital equipment and the use of social networks), sociodemographic characteristics (age, gender, socioprofessional category), geographical characteristics (determined by the postcode), and the medical characteristics (medication and long-term disease). The socioprofessional categories were derived from the nomenclature of the French National Institute for Statistical and Economic Studies (Institut National de la Statistique et des Études Économiques [INSEE]) and they were gathered in four categories (two for the labor force and two for the nonworking population) by median annual income. The types of used health apps were derived from the French National Market Research Agency (Institut Français d’Opinion Publique [IFOP]) and simplified to be more comprehensible. The following variables regarding the type of health app use were collected: treatment and follow-up care (4 classes); emergency management (2 classes); communication (3 classes); well-being, prevention, and fitness (5 classes); fertility and pregnancy (2 classes); and diagnostic assistance (2 classes) (see Table 1).

**Table 1:** Types of health apps.

Category	Subclass
Treatment and follow-up care	Drug information, disease information, medical parameters management (eg, blood pressure, weight), help receiving treatments
Emergency management	Warning system (eg, prerecorded emergency numbers), first-aid help (eg, basic emergency life-saving skills)
Communication	Health professional search (eg, phone books), data sharing (eg, medical mailbox), exchange about health themes
Well-being, prevention, and fitness	Smoking cessation, fitness, nutrition and weight loss, stress management, sleeping help
Fertility and pregnancy	Ovulation schedule, pregnancy calendar
Diagnostic assistance	Symptom information, self-diagnosis help

### Data Analysis

The database input was performed by two authors, and entries were verified for 10% of the questionnaires. Statistical analyses were performed with Stata 15.0. Quantitative variables are expressed as mean (SD) and were compared with a *t*-test after normality was confirmed or with the Mann-Whitney *U* test if normality was not confirmed. Qualitative variables are expressed in numbers with percentages and were compared with the *χ*^2^ test (or Fisher exact test in the event of small numbers). Statistical testing was performed with an α risk equal to .05. Multivariate analysis was performed by logistic regression with a subgroup analysis. Variables selected were those with *P*<.20 in univariate analysis. Odds ratios (ORs) were calculated with the 95% CIs for each variable. Missing data were incorporated in the statistical analysis.

### Ethics Approval and Registration

This study was approved by the Committee for the Protection of Persons (Comité de Protection des Personnes) Sud-Ouest et Outre-Mer III (2017-A01647-46) and by the Correspondent for Protection of Personal Data (Correspondant Informatique et Libertés) of University Grenoble Alps (0987763). The protocol was registered at ClinicalTrials.gov (NCT03351491).

## Results

The response rate of the distributed questionnaires was estimated at 49.3% (739/1500; 95% CI 46.7%-51.8%). Eight patients refused to give their consent (Table 2). Among our study participants, the proportion of mobile phone owners was estimated at 96.6% (714/731; 95% CI 96.3%-98.5%). Smartphone use was estimated at 82.6% (597/723; 95% CI 79.6%-85.2%). A total of 134 patients were excluded from the final analysis: 119 did not use a smartphone, 7 did not know what kind of mobile phone they used, and 8 questionnaires contained missing data concerning the use of a smartphone. A total of 597 questionnaires were then included in the statistical analysis.

The sample characteristics are summarized in Table 3. The average age of the patients was 41.1 (SD 5.6) years and 65% of the sample were women (95% CI 61.1%-68.7%).

Most of the participants (455/594, 76.6%; 95% CI 73.0%-79.8%) used apps on their smartphone, and 47.7% (283/595; 95% CI 43.6%-51.6%) used at least one health app since acquiring their smartphone. All of these users (283/283, 100.0%) had already downloaded an app (health app or not) on apps markets. The most commonly used app types were well-being, prevention, or fitness apps (185/280, 66.1%; 95% CI 60.3%-71.4%); apps about drugs, treatment, and follow-up care (140/280, 50.0%; 95% CI 44.2%-56.0%); apps for communication with health professionals (98/280, 35.0%; 95% CI 29.6%-40.8%); and apps about fertility and pregnancy (58/280, 20.7%; 95% CI 16.3%-25.9%).

The least used apps were those for emergency management (27/280, 9.6%; 95% CI 6.9%-13.7%) and apps for diagnosis help and symptoms data (26/280, 9.3%; 95% CI 6.4%-13.3%). The use of more than one app was reported by 46.6% (132/280; 95% CI 40.9%-52.5%) of patients. Women used more health apps about fertility and pregnancy; well-being, prevention, and fitness; and drugs, treatment, and follow-up care than men (Table 4).

The main origins of awareness of the existence of the used apps were the social circle (114/265, 43%; 95% CI 37.1%-49.1%) and media (internet, TV, newspapers, and other media channels) (103/265, 38.9%; 95% CI 33.1%-44.9%). In addition, 8.7% (23/265; 95% CI 5.8%-12.7%) of GPs recommended health apps to patients, whereas only 6.0% (16/265; 95% CI 3.7%-9.6%) and 3.7% (10/265; 95% CI 2.0%-6.9%) of other physicians and other health professionals, respectively, recommended these apps. Other sources were given for 19.2% (51/265; 95% CI 14.9%-24.5%) of the respondents, including preinstalled health apps (10/39) or downloaded apps following personal research (11/39). The frequency of use of these apps was indicated to be rare for 31.1% (84/270; 95% CI 25.8%-36.9%), monthly for 21.5% (58/270; 95% CI 16.9%-26.8%), weekly for 16.3% (44/270; 95% CI 12.3%-21.2%), and daily for 31.1% (84/270; 95% CI 25.8%-36.9%) of respondents.

Univariate analysis revealed that the use of health apps was associated with a young population, mainly female, living in larger cities, and the use of social network(s) (all *P*<.001). A potential association between health app use and socioprofessional category (*P*<.001) was also identified.

By contrast, the presence of chronic conditions and the number of treatments were not associated with the use of health apps (Table 5).

The logistic regression model displayed an association between the use of health apps and the use of social networks; age under 30 years; being a woman; living in a city with 5001-10,000 inhabitants; living in a city with more than 10,000 inhabitants; and occupying an executive position, intellectual profession, or having an intermediate occupation (Table 6).

**Table 2:** Participation by center.

Center	Distributed (N=1500)	Collected (n=739), n (%)	Participants (% total sample)	Women (n=473), n (%)	Refusal to participate, n (%)	Smartphone user (n=597), n (%)
1	43	13 (30.2)	1.76	7 (1.5)	0 (0)	10 (1.7)
2	172	43 (25.0)	5.82	26 (5.5)	2 (25)	31 (5.2)
3	129	62 (48.1)	8.39	40 (8.5)	0 (0)	52 (8.7)
4	172	127 (73.8)	17.19	87 (18.4)	0 (0)	107 (17.9)
5	43	33 (76.7)	4.47	27 (5.7)	0 (0)	30 (5.0)
6	43	38 (88.4)	5.14	26 (5.5)	1 (12.5)	36 (6.0)
7	167	96 (57.5)	12.99	59 (12.5)	3 (37.5)	80 (13.4)
8	129	122 (94.6)	16.51	80 (16.9)	0 (0)	94 (15.7)
9	172	94 (54.7)	12.72	47 (9.9)	0 (0)	68 (11.4)
10	86	45 (52.3)	6.09	28 (5.9)	1 (12.5)	35 (5.9)
11	43	29 (67.4)	3.92	21 (4.4)	1 (12.5)	24 (4.0)
12	43	19 (44.2)	2.57	13 (2.7)	0 (0)	16 (2.7)
13	258	18 (7.0)	2.44	12 (2.5)	0 (0)	14 (2.3)

**Table 3:** Characteristics of the sample.

Characteristic		Value
**Age (years) (n=596), n (%)**		
	<30	143 (24.0)
	30-39	140 (23.5)
	40-49	134 (22.5)
	50-69	179 (30.0)
Women (n=597), n (%)		388 (65.0)
Use of social network(s) (n=597), n (%)		464 (72.7)
**Chronic disease, n (%)**		
	Overall (n=592)	204 (34.5)
	Cancer (n=575)	18 (3.1)
	Diabetes (n=577)	28 (4.8)
	Psychiatric diseases (n=577)	19 (3.3)
	Cardiac diseases (n=577)	36 (6.7)
	Rheumatologic diseases (n=577)	29 (5.0)
	Pulmonary diseases (n=577)	14 (2.4)
	Renal diseases (n=577)	5 (0.9)
	Other chronic diseases (n=577)	80 (13.9)
Medications (n=583) (number per day), mean (SD)		0.9 (0.7)
**Population (n=597), n (%)**		
	0-1000	7 (1.2)
	1001-5000	190 (31.8)
	5001-10,000	177 (29.6)
	>10,000	223 (37.3)
**Socioprofessional category (n=595), n (%)**		
	Farmers, craftspeople, storekeepers, managers, workers, or employees	295 (49.6)
	Executive, intermediate, or intellectual professions	159 (26.7)
	Retired	52 (8.7)
	Student or unemployed	89 (15.0)

**Table 4:** Use and type of health apps used by gender.

Use of health app	Respondents, n	Women, n (%)	Men, n (%)	*P* value
Overall users	283	206 (73.0)	77 (27.0)	<.001
Drugs, treatment, and follow-up care	140	102 (72.9)	38 (27.1)	<.001
Emergency management	27	17 (63.0)	10 (37.0)	.84
Communication with health professionals	98	69 (70.4)	29 (29.6)	.25
Well-being, prevention, and fitness	185	134 (72.4)	51 (27.6)	<.001
Fertility and pregnancy	58	57 (98.3)	1 (1.7)	<.001
Diagnosis help and symptoms data	26	21 (80.8)	5 (19.2)	.10

**Table 5:** Univariate analysis of characteristics associated with the use of health apps.

Characteristic			Health apps use (n=283)		No health apps use (n=312)		*P* value	
Age (years) (n=596), mean (SD)			37.7 (7.8)		44.2 (7.7)		<.001	
Women (n=388), n (%)			206 (72.8)		180 (57.7)		<.001	
Use of social network(s) (n=594), n (%)			244 (86.2)		190 (60.9)		<.001	
**Chronic disease** **, n (%)**								
	Overall (n=592)	90 (32.0)		114 (36.9)		.12		
	Cancer (n=575)	10 (3.6)		8 (2.7)		.35		
	Diabetes (n=577)	10 (3.6)		18 (6.0)		.12		
	Psychiatric diseases (n=577)	10 (3.6)		9 (3.0)		.44		
	Cardiac diseases (n=577)	14 (5.1)		22 (7.4)		.16		
	Rheumatologic diseases (n=577)	16 (5.8)		13 (4.4)		.28		
	Pulmonary diseases (n=577)	7 (2.5)		7 (2.4)		.55		
	Renal diseases (n=577)	3 (1.1)		2 (0.7)		.47		
	Other chronic diseases (n=577)	39 (14.1)		41 (13.8)		.50		
Number of treatments (n=583), mean (SD)			0.9 (0.1)		1.0 (0.1)		.23	
**Population (n=596), n (%)**								<.001
	0-1000	1 (0.3)		6 (1.9)				
	1001-5000	69 (24.4)		121 (38.8)				
	5001-10,000	89 (31.4)		87 (27.8)				
	>10,000	124 (43.8)		98 (31.4)				
**Socioprofessional category (n=595), n (%)**								.001
	Farmers, craftspeople, storekeepers, managers, workers or employees	141 (50.0)		152 (48.9)				
	Executives, intermediate or intellectual professions	83 (29.4)		76 (24.4)				
	Retired	13 (4.6)		39 (12.5)				
	Students or unemployed	45 (16.0)		44 (14.1)				

**Table 6:** Factors associated with the use of health apps (logistic regression model).

Characteristic			Adjusted OR^a^		95% CI
**Age (years)**					
	18-29 (n=143)	2.68		1.45-4.94	
	30-39 (n=139)	1.22		0.70-2.15	
	40-49 (n=134)	1.46		0.85-2.51	
	50-69 (n=178)	Reference		Reference	
**Socioprofessional category**					
	Farmers, craftspeople, storekeepers, managers, workers, or employees	Reference		Reference	
	Executives, intermediate or intellectual professions	1.71		1.10-2.65	
	Retired	0.80		0.36-1.80	
	Students or unemployed	0.76		0.45-1.29	
Women			1.77		1.21-2.59
Chronic disease			1.28		0.83-1.96
**Population**					
	0-5,000	Reference		Reference	
	5001-10,000	1.81		1.15-2.84	
	>10,000	2.10		1.37-3.22	
Social network(s) use			3.36		2.12-5.34

^a^OR: odds ratio.

## Discussion

### Principal Findings

In this survey, 82.6% of participants owned a smartphone, 76.6% used apps, and 46.7% used at least one health app. These users of health apps tended to be under 30 years old, women, use social networks, belong to a higher socioprofessional category, and live in larger cities compared with other smartphone owners. Over 84% of the population over 12 years old in France was reported to use a smartphone in 2020 (representing an increase from 77% in 2019) [13], with 85% of US adults estimated to use a smartphone in 2020 (representing an increase from 81% in 2019) [14]. The smartphone user proportion of 82.6% (597/723; 95% CI 79.6%-85.2%) found in this study was thus slightly higher than the estimate of French data from 2019. This difference could be explained by the characteristics of our sample with a young, tech-friendly population. Indeed, we did not include people over 69 years old. As this older population is less equipped in smartphones than people between 18 and 69 years, not including them should have caused a higher proportion of smartphone owners in our sample.

No estimation of the use of health apps in France was found in the scientific literature. Therefore, the results of this survey had to be compared to those of other countries. To our knowledge, the more recently published results about the rate of use of general health apps concern the United States and Germany, with both studies conducted in 2015, along with one study conducted in Hong Kong in 2016 [12], providing estimates of health apps use among smartphone owners of 20.5% [15], 58.2% [10], and 24.1% [12], respectively. These differences could be attributed to the different sample recruitment strategies used. Our sample was recruited from GPs in their office or in a primary care center, whereas the previous studies were conducted through population-based surveys. Thus, selection bias may be possible, because health consciousness might be more important among patients than among the general population [16].

This disparity could be also attributed to differences in sample characteristics, and especially the mean age. Indeed, our studied sample had a mean age of 41 years, which is close to that of the US survey (40 years); however, the mean age of the sample in Germany was 57 years and 16.1% of the participants of the Hong Kong survey were elderly patients (≥65 years). Our results suggested that the younger population had a greater tendency to use health apps than older participants. In addition, a recent systematic review regarding factors influencing use of mHealth apps showed that mHealth apps are mainly used by young people [17]. Thus, by excluding patients over 69 years of age in our survey, the mean age decreased while the overall use of health apps increased in comparison with those reported for the general population.

In our results, the most frequently used health apps were those concerning well-being, prevention, and fitness (185/280, 66.1%), and those related to treatment, drugs, and follow-up care (140/280, 50.0%). These could not be directly compared to the previous studies of Ernsting et al [11] and Krebs and Duncan [10], as they measured the reasons for downloading apps but did not directly compare the types of apps downloaded. In the German survey [11], the participants mainly reported using apps to support changes in smoking cessation (44.5%), healthy diet (38.6%), weight loss (23.2%), and physical activity (17.08%), whereas the participants of the US survey [10] reported physical activity tracking (52.8%), nutrition tracking (47.6%), and the desire to lose weight (46.8%) as the main reasons to download health apps. The results of the Hong Kong survey [12] also showed that physical activity tracking (67%), logging health records (43%), and tracking health measures (30.2%) were the most frequent reasons for downloading and using apps. Despite the differences of measurement methods, these data seem to reinforce our results and confirm a greater interest for prevention, fitness, and well-being apps. This same conclusion was reached by the Pew Research Center in 2012 in that health apps related to fitness (38%), diet (31%), and weight management (12%) were more common, whereas medication management apps were only used by 2% of respondents in a US survey [18].

Concerning the frequency of health apps use, this study found either rare (84/270, 31.1%) or daily use (84/270, 31.1%) as the most common answers. These opposite results might be explained by the gradual loss of interest for the app(s) over time; patients likely use their apps more often initially due to the appeal of novelty, which then decreases progressively. This hypothesis was also supported by the US survey [10], in which 45.7% of health app users no longer used the health app, including 40% who indicated that this was due to a loss of interest.

Our results demonstrated an association of being aged under 30 years with the use of mHealth apps. The same conclusions were obtained in other surveys. This association could be explained by the high smartphone ownership rate among younger patients and their tendency to download more apps; 80% among those 18-24 years of age and 72% among those 25-39 years of age used their mobile phones to download app(s) in France compared with only 44% among those 40-59 years of age and 20% among those 60-69 years of age [9].

Social networks use was also associated with the use of health apps on a smartphone (OR 3.36, 95% CI 2.1-5.3). We can suppose that this is because social network users are more inclined to download apps in general, and thus more health apps, than nonusers.

We also found an association of health app use with gender. Indeed, women appeared to use health apps more than men. This might be due to the use of fertility and pregnancy apps. This association could be qualified because this link was not demonstrated in previously reported surveys, and because women seemed to be overrepresented in our sample (65%, 95% CI 61.1%- 68.7%). However, the previous surveys were conducted in the general population, whereas ours focused on people consulting a GP. Women tend to consult GPs more often than men, especially before 55 years of age, and therefore the characteristics of our sample could explain this difference. Moreover, the same association was reported in the Pew Research Center survey [18].

Our results also suggested that people who occupied an executive position, an intellectual profession, or an intermediate occupation had a tendency to use health apps more than others. This could be partly explained by the higher use of smartphones among people in this socioprofessional category and by their greater use of apps in general. An association was also found between health apps use, a high income, and higher level of education in previous surveys [13,15]. This therefore strengthens our conclusions, as the identified socioprofessional category comprises those earning the most money and having followed the highest level of education.

Moreover, living in cities with more than 5000 inhabitants was also associated with greater use of health apps. Indeed, the multivariate analysis demonstrated that the bigger the city, the stronger the association. Thus, cities with more than 10,000 inhabitants were more strongly associated with apps use (OR 2.10, 95% CI 1.37-3.22) than those with 5001-10,000 inhabitants (OR 1.81, 95% CI 1.15-2.84). The size of cities was not explored in other surveys. These data might be explained by the concentration of students in Grenoble (8.0% vs 2.0% in the same region in 2010), who are younger and thus more likely to use apps, and of higher-income people such as executives or those with intermediate occupations (14.0% and 16.6%, respectively, vs 11.9% and 16% in the whole region) in these areas.

In contrast to the findings of Ernsting et al [11], we did not find an association of health apps use with having a chronic disease. This result could be explained by our inclusion criteria. Indeed, patients over 69 years old, who represent the population most affected by chronic diseases, were not included in our sample. Moreover, we did not consider BMI or ethnicity in our study, which were associated with health apps use in the other surveys. A survey in the general population or without limitation of age would be interesting to further study the associations of these factors.

### Limitations

Despite the agreement of these results with previous data from the literature, some limitations should be taken into consideration with respect to interpreting the results of this study. First, this study was not randomized and the participation was voluntary, which could be a limitation of sample representativeness. Furthermore, we did not explore the use of health apps in the population over 60 years old. This choice of inclusion criterion might have created a bias in assessing the associations with chronic disease and the number of treatments, which were found in some previous studies. Finally, this was a self-response survey, which can cause misunderstandings and missing data for some questions in the questionnaire, despite performing pilot tests with several patients before official recruitment. Considering these limitations, these results from a regional survey are consequently hard to extend to the general population.

This study was performed prior to the COVID-19 pandemic. During the pandemic period, eHealth, mHealth, and telemedicine were more widely adopted for crisis management and as a preventive measure to increase clinical care [19,20]. Thus, a similar study should be performed to determine the impact of the pandemic on the use of eHealth. An additional element in this survey was that there were no questions related to the use of health apps by GPs; thus, it would be interesting to evaluate the potential link between this practice and the patients’ use of mHealth.

In spite of these limitations, precautions were taken to ensure the relevance of the results. The sample was composed of patients from 13 centers that represented various types of primary care practices. The substantial sample size further instilled confidence in the robustness of the statistical methods used, with sufficient power to detect statistically significant differences. Moreover, we used paper questionnaires rather than online surveys. This decision was taken so as to avoid selection bias of people that use the internet regularly, who would also be more likely to own a smartphone and to use health apps. These precautions were used to ensure the strength of our findings, and our results indeed are in agreement with studies performed in other countries.

### Conclusion

This work confirmed the wide extent of smartphone use among 82.6% of the sample. Moreover, an important use of health apps was identified in our sample, with nearly one out of two smartphone users reporting downloading and using health apps. The users of these apps had a tendency to be younger than 30 years old, to be women, to live in bigger cities, to use more social networks, and to be in a high socioprofessional category. Currently, the use of mHealth is mainly limited to well-being and prevention apps, including fitness and weight loss apps. However, health apps have demonstrated their potential in changing health behavior and could thus become a new tool for health professionals to help improve their patients’ health conditions. Physicians could design health apps or advise patients about them, aiming to improve their relationship and facilitating the development of partnership in their care. Nevertheless, health apps could be more efficient if they reached a broader population. Making these apps easier to use could also help to democratize them. Accordingly, health apps could play a major role in health care, especially for people affected by chronic conditions or for elderly people. App developers are therefore encouraged to improve their software to make their apps more accessible to a maximum of patients. However, the development of health apps and the huge and increasing number of app marketplaces come with a problem of reduced quality; indeed, important discrepancies can be observed between apps, and patients do not currently have the appropriate information and formal evidence of their effectiveness. This major challenge is currently under discussion in France and worldwide [21].

## Appendix

Multimedia Appendix 1 Details of health centers participating in the study.

## Abbreviations

CNEDIMTS: National Committee for the Evaluation of Medical Devices and Health Technologies
GP: general practitioner
IFOP: Institut Français d’Opinion Publique (French National Market Research Agency)
INSEE: Institut National de la Statistique et des Études Économiques (French National Institute for Statistical and Economic Studies)
mHealth: mobile health
OR: odds ratio

## References

[1] Ryu S (2012). Book Review: mHealth: New Horizons for Health through Mobile Technologies: Based on the Findings of the Second Global Survey on eHealth (Global Observatory for eHealth Series, Volume 3). Healthc Inform Res.

[2] Barometre du numerique edition 2021. Enquête sur la diffusion des technologies de l’information et de la communication dans la société française. ARCEP.

[3] Perrin A (2021). Mobile technology and home broadband. Pew Research Center.

[4] Han M, Lee E (2018). Effectiveness of mobile health application use to improve health behavior changes: a systematic review of randomized controlled trials. Healthc Inform Res.

[5] Marcolino MS, Oliveira JAQ, D'Agostino M, Ribeiro AL, Alkmim MBM, Novillo-Ortiz D (2018). The impact of mHealth interventions: systematic review of systematic reviews. JMIR Mhealth Uhealth.

[6] Müssener U (2021). Digital encounters: human interactions in mHealth behavior change interventions. Digit Health.

[7] Vo V, Auroy L, Sarradon-Eck A (2019). Patients' perceptions of mHealth apps: meta-ethnographic review of qualitative studies. JMIR Mhealth Uhealth.

[8] Sarradon-Eck A, Bouchez T, Auroy L, Schuers M, Darmon D (2021). Attitudes of general practitioners toward prescription of mobile health apps: qualitative study. JMIR Mhealth Uhealth.

[9] Hassanaly P, Dufour JC (2021). Analysis of the regulatory, legal, and medical conditions for the prescription of mobile health applications in the United States, the European Union, and France. Med Devices (Auckl).

[10] Krebs P, Duncan DT (2015). Health app use among US mobile phone owners: a national survey. JMIR Mhealth Uhealth.

[11] Ernsting C, Stühmann LM, Dombrowski SU, Voigt-Antons J, Kuhlmey A, Gellert P (2019). Associations of Health App Use and Perceived Effectiveness in people with cardiovascular diseases and diabetes: population-based survey. JMIR Mhealth Uhealth.

[12] Shen C, Wang MP, Chu JT, Wan A, Viswanath K, Chan SSC, Lam TH (2017). Health app possession among smartphone or tablet owners in Hong Kong: population-based survey. JMIR Mhealth Uhealth.

[13] Baromètre du numérique 2019. Enquête sur la diffusion des technologies de l’information et de la communication dans la société française en 2019. ARCEP.

[14] Anderson M (2019). Mobile technology and home broadband 2019. Pew Research Center.

[15] Ernsting C, Dombrowski SU, Oedekoven M, O Sullivan JL, Kanzler M, Kuhlmey A, Gellert P (2017). Using smartphones and health apps to change and manage health behaviors: a population-based survey. J Med Internet Res.

[16] Jayanti RK, Burns AC (1998). The antecedents of preventive health care behavior: an empirical study. J Acad Market Sci.

[17] Wang C, Qi H (2021). Influencing factors of acceptance and use behavior of mobile health application users: systematic review. Healthcare (Basel).

[18] Fox S, Duggan M (2012). Mobile health 2012. Pew Research Center.

[19] Petrazzuoli F, Kurpas D, Vinker S, Sarkisova V, Eleftheriou A, Żakowicz A, Aarendonk D, Ungan M (2021). COVID-19 pandemic and the great impulse to telemedicine: the basis of the WONCA Europe Statement on Telemedicine at the WHO Europe 70th Regional Meeting September 2020. Prim Health Care Res Dev.

[20] Bitar H, Alismail S (2021). The role of eHealth, telehealth, and telemedicine for chronic disease patients during COVID-19 pandemic: A rapid systematic review. Digit Health.

[21] Mathews SC, McShea MJ, Hanley CL, Ravitz A, Labrique AB, Cohen AB (2019). Digital health: a path to validation. NPJ Digit Med.

