# Supplementary material for: Use of Smartphone Health Apps Among Patients Aged 18 to 69 Years in Primary Care: Population-Based Cross-sectional Survey
Source: JMIR Form Res. 2022 Jun 16;6(6):e34882. doi: 10.2196/34882 (PMC9247815; doi:10.2196/34882)
Supplement: Multimedia Appendix 1 [file formative_v6i6e34882_app1.docx]

| Center | Numbers of GP participant | Other health professionals in the center (no participant) | Type of practice | City (number of population) |
| --- | --- | --- | --- | --- |
| 1 | 1/1 | No | Self-employed | Châbon (2,100) |
| 2 | 4/4 | No | Self-employed | Villard de Lans (4,143) |
| 3 | 3/3 | No | Self-employed | Montmelian (4,100) |
| 4 | 4/6 | Physiotherapist, nurses, dentist | Self-employed | Tullins (7,650) |
| 5 | 1/2 | No | Self-employed | Grenoble (160,650) |
| 6 | 1/3 | No | Self-employed | Meylan (17,290) |
| 7 | 4/4 | No | Employed | Grenoble (160,650) |
| 8 | 3/3 | No | Self-employed | La Côte saint-André (4,750) |
| 9 | 4/6 | Allergist, Podiatrist, Midwife | Self-employed | Moirans (8,000) |
| 10 | 2/2 | No | Self-employed | Saint-Martin-D’Hères (38,500) |
| 11 | 1/3 | No | Self-employed | Grenoble (160,650) |
| 12 | 1/3 | No | Self-employed | Grenoble (160,650) |
| 13 | 6/6 | No | Self-employed | Saint-Martin-D’Hères (38,500) |
